# Exposure to Nanoplastics During Pregnancy Induces Brown Adipose Tissue Whitening in Male Offspring

**DOI:** 10.3390/toxics13030171

**Published:** 2025-02-27

**Authors:** Zhaoping Shen, Kai Tian, Jiayi Tang, Lin Wang, Fangsicheng Zhang, Lingjuan Yang, Yufei Ge, Mengna Jiang, Xinyuan Zhao, Jinxian Yang, Guangdi Chen, Xiaoke Wang

**Affiliations:** 1 Nantong Key Laboratory of Environmental Toxicology, Department of Occupational Medicine and Environmental Toxicology, School of Public Health, Nantong University, Nantong 226019, China; shenzp0324@163.com (Z.S.); tkai14188@gmail.com (K.T.); 2317320005@stmail.ntu.edu.cn (J.T.); wlin0223@163.com (L.W.); z17799389232@163.com (F.Z.); 2417310032@stmail.ntu.edu.cn (L.Y.); geyufei917@163.com (Y.G.); 17851790126@163.com (M.J.); zhaoxinyuan@ntu.edu.cn (X.Z.); 2 Xinglin College, Nantong University, Qidong 226236, China; yjx0815@ntu.edu.cn; 3 Department of Public Health, Zhejiang University School of Medicine, Hangzhou 310058, China

**Keywords:** polystyrene nanoplastics, pregnancy, obesity, brown adipose tissue whitening, lipogenesis, lipophagy

## Abstract

Background: Polystyrene nanoplastics (PSNPs) have been recognized as emerging environmental pollutants with potential health impacts, particularly on metabolic disorders. However, the mechanism by which gestational exposure to PSNPs induces obesity in offspring remains unclear. This study, focused on the whitening of brown adipose tissue (BAT), aims to elucidate the fundamental mechanisms by which prenatal exposure to PSNPs promotes obesity development in mouse offspring. Methods and Results: Pregnant dams were subjected to various doses of PSNPs (0 µg/µL, 0.5 µg/µL, and 1 µg/µL), and their offspring were analyzed for alterations in body weight, adipose tissue morphology, thermogenesis, adipogenesis, and lipophagy. The findings revealed a notable reduction in birth weight and an increase in white adipocyte size in adult offspring mice. Notably, adult male mice exhibited BAT whitening, correlated with a negative dose-dependent downregulation of UCP1 expression, indicating thermogenesis dysfunction. Further investigation revealed augmented lipogenesis evidenced by the upregulation of FASN, SREBP-1c, CD36, and DGAT2 expression, coupled with the inhibition of lipophagy, indicated by elevated levels of mTOR, AKT, and p62 proteins and reduced levels of LC3II/LCI and Lamp2 proteins in male offspring. Conclusions: These findings indicate that gestational PSNP exposure plays a role in the development of obesity in offspring through the whitening of brown adipose tissue, which is triggered by lipogenesis and lipophagy inhibition, providing a novel insight into the metabolic risks associated with gestational PSNPs exposure.

## 1. Introduction

Plastics have attracted enormous attention for their wide applications across various fields, including industrial, technical, medical, cosmetic, and life sciences [1], and their presence in the environment is increasing at an alarming rate [2]. Microplastics (MPs, 100 nm–5 mm) and nanoplastics (NPs, <100 nm) originate from the degradation of discarded plastics through mechanical weathering, ultraviolet radiation, and biological processes [3]. Microplastics and nanoplastics (MNPs) are extensively found in freshwater, soil, and the atmosphere [4]. Generally, MNPs enter the body via contaminated water and food, while in areas where synthetic materials are prevalent, recent studies have also demonstrated that the atmosphere also serves as an important medium for transportation [5]. And nanoplastics have emerged as a novel environmental threat due to their small size and enhanced capacity to penetrate cells, tissues, and even placentas [6,7].

With reports of microplastics in humans increasing, the potential health threats they pose are gradually gaining attention. Previous studies [8,9] elucidated that MNPs may cause physical, chemical, and microbial toxicity, leading to oxidative stress, inflammation, immune responses, DNA damage, and other health impacts. An increasing number of studies are showing that exposure to nanoplastics causes lipid metabolism disorders. Exposure to polystyrene nanoplastics (PSNPs) markedly elevates the expression levels of genes pertinent to lipid metabolism in the liver of *Dicentrarchus labrax* [10]. PSNPs also enhance the expression of genes involved in lipid synthesis (FASN and CD36) while diminishing the expression of genes linked to lipid catabolism (ATGL and PPAR-α) [11]. Lipid metabolism disorders contribute to obesity, which is a significant public health issue, with a prevalence continuously rising worldwide [12]. From 1980 to 2015, the prevalence of obesity doubled in 73 countries, with most other countries also experiencing an increase [13]. By 2030, it is projected that 38% of the world’s adults will be overweight, and an additional 20% will be obese, placing a heavy burden on both human health and the economy [14].

The theory of the Developmental Origins of Health and Disease (DOHaD) suggests that prenatal adverse environmental factors can have a substantial influence on offspring’s long-term health, including with respect to metabolic diseases [15]. Several studies have indicated that there is a causal relationship between the occurrence of adult offspring obesity and hazardous substances such as BPA PM_2.5_ and smoking [16,17,18]. In a mouse model, an investigation revealed altered serum glycolipid metabolism, which may contribute to abnormal weight gain in adulthood following maternal polystyrene MP exposure during gestation and lactation [19]. Additionally, oral intake of nanoplastics by mothers was found to be associated with the early postnatal weight of offspring, especially male offspring [20]. Concurrently, our previous study [21,22] demonstrated that the prenatal exposure of mice to airborne PSNPs led to weight gain in the adult offspring. Collectively, these studies suggest that early-life exposure to PSNPs can trigger the development of obesity in offspring. Nevertheless, the underlying mechanisms remain elusive.

It is widely recognized that BAT plays a pivotal role in augmenting energy expenditure via thermogenesis [23]. This thermogenic process is regulated by uncoupling protein 1 (UCP1), which uncouples the oxidative respiratory chain within mitochondria [23,24]. However, when BAT undergoes a process known as “whitening”, characterized by the accumulation of lipid droplets, the result is a decline in energy metabolism. The unique thermogenic ability of BAT renders it a crucial inducible target for exploring the pathogenesis of obesity. This is particularly significant given the confirmation of BAT’s presence in adults through positron emission tomography (PET) scans [25]. Prior research has indicated that there is a strong association between BAT whitening and diet-induced obesity [26]. Moreover, interventions involving, e.g., dietary lactate intake and physical exercise, which can reverse BAT whitening, have been shown to ameliorate diet-induced obesity [27]. Based on these findings, we hypothesize that BAT whitening mediates the development of offspring obesity induced by prenatal exposure to PSNPs. In this study, we employed a rodent model to investigate the impact of gestational PSNPs exposure on BAT whitening in offspring and elucidate the underlying mechanisms driving this process.

## 2. Materials and Methods

### 2.1. Materials

The PSNPs suspension (particle size: 80 nm) was purchased from the Beisile Chromatography Technology Development Center (Tianjin, China; Catalog Number: 6-1-0005), as previously used, with an average size of 71.09 ± 6.63 nm and spherical in shape [21]. Before preparing various concentrations, the suspension was ultrasonically dispersed for 30 min and then diluted with sterile phosphate-buffered saline (pH = 7.2). Each dilution was followed by 15 min of ultrasonication to ensure optimal particle dispersion. The prepared suspensions were stored at 4 °C and ultrasonicated for 15 min before use.

### 2.2. Experimental Animals

Virginal 10-week-old C57BL/6J mice (SPF grade) were purchased from Nantong University Laboratory Animal Center. All mice were accommodated in animal facilities under a standard 12 h: 12 h light/dark regimen, with a temperature maintained at 23 ± 1 °C and humidity at 55 ± 10%, while food and water were provided ad libitum.

### 2.3. Animal Model Development

After a one-week acclimatization period, female and male mice were co-housed overnight in a 2:1 ratio. We confirmed pregnancy via the presence of a vaginal plug the next day. Thirty pregnant mice were divided into control (C), low-dose (L), and high-dose (H) groups (10 mice/group) randomly, based on the PSNPs exposure levels from previous studies [21]. The control group was administered 0 µg/µL (0 particles/day), the low-dose group was administered 0.5 µg/µL (about 0.15 × 10^11^ particles/day), and the high-dose group was administered 1 µg/µL (about 0.30 × 10^11^ particles/day). Mice were exposed to 50 µL of the suspension via oropharyngeal aspiration every other day until delivery. After delivery, litter size, sex ratio, and body weight were recorded. In order to avoid nutritional and sex bias, we randomly culled the pups to 6–8 per litter upon birth and retained equal male-to-female ratios. Following weaning at postnatal week 4, offspring were grouped based on prenatal exposure dosage and sex, establishing three experimental groups per gender, namely, female control (FC), low-dose (FL), and high-dose (FH) groups, with corresponding male groups designated as MC, ML, and MH. At 15 weeks of age, following a 12 h fasting period, the offspring were humanely euthanized. After euthanasia, the gonadal white adipose tissue (WAT) and interscapular brown adipose tissue (BAT) were dissected and weighted, and the organ coefficients were calculated as wet organ weight (WAT/BAT)/body weight*100%. The Animal Care and Use Committee of Nantong University approved all procedures.

### 2.4. Histological Analysis

White and brown adipose tissue from randomly selected offspring per group were fixed in 10% neutral formalin for 24 h, dehydrated using a graded ethanol series (70%, 80%, 90%, 95%, and absolute ethanol), embedded in paraffin, sectioned (5 μm), and stained with hematoxylin and eosin (H&E). For each mouse, six tissue sections were prepared for analysis. For the analysis of adipocyte size, we randomly chose 2–3 slices from each of the six sections per animal for quantification. We examined at least three different regions per section using a standard light microscope. The quantification of adipocyte cell size was carried out using Image J software (version 1.53t, NIH, Bethesda, MD, USA), and the results were normalized to the control group. The obtained data were then normalized to the control group for subsequent analysis. At least two experts performed pathological evaluation.

### 2.5. mRNA Level Analysis

Total RNA was extracted from brown adipose tissue using the Trizol (Takara, Beijing, China) method [28]. We validated primer efficiency by serially diluting a standard sample (cDNA) and performing qPCR. A standard curve was generated by plotting the dilution factor against the Ct values. We considered primer efficiency acceptable if it fell within the range of 90–110% and the melt curve showed a single peak [29]. The extracted RNA was reverse-transcribed into cDNA using M-MLV reverse transcriptase, 10 µM of dNTP, oligo(dT) primers, and DEPC-treated water [30]. Prior to quantitative analysis, primer efficiency was validated using RNA extracted from mouse adipose tissue along with negative control samples to ensure specificity and efficiency for the intended targets [29]. We also established negative controls, including no-template controls (NTCs), for which pure water was used instead of the nucleic acid template. For this control group, no amplification signal indicates there is no contamination. NTCs were included in all PCR reactions to prevent contamination and ensure reliability [29]. Quantitative real-time PCR (qPCR) was performed using a Light Cycler system (Roche, Germany) with a final reaction volume of 10 µL, composed of 3.2 µL of RNA-free water, 5 µL of TB Green, 0.4 µL of forward primer, 0.4 µL of reverse primer, and 1 µL of cDNA. For each experimental condition, six biological replicates and three technical replicates were used. β-actin served as the reference gene, and the 2^ΔΔCt^ method was employed to assess relative mRNA levels [30]. Gene primers are listed in Table 1.

### 2.6. Western Blotting

Total protein was extracted from brown adipose tissue using RIPA lysis buffer (Beyotime, Shanghai, China) on ice. Protein concentrations were determined using the BCA assay (Beyotime, Shanghai, China). The samples were then prepared at equal concentrations and volumes for subsequent analysis. As previously reported [31], SDS-PAGE was used to separate proteins by applying a constant voltage of 80 V for 90 min. After electrophoresis, proteins were transferred to PVDF membranes (Millipore, Bedford, MA, USA) using a wet-transfer method at a constant current of 400 mA for 70 min under ice bath conditions. The membranes were blocked with a protein-free rapid blocking buffer (Affinibody, Wuhan, Hubei, China) at 4 °C for at least 15 min. After the blocking stage, the membranes were incubated with primary antibodies (as shown in Table 2.) at 4 °C and, subsequently, secondary antibodies at room temperature. After primary and secondary antibody incubations, the membranes were washed at least three times for 10–15 min each with pre-chilled TBST to remove non-specific signals. Protein bands were visualized using chemiluminescence under the same exposure conditions, and signal intensity was measured using ImageJ (version 1.53t, NIH, Bethesda, MD, USA) software, with background signals subtracted. For each dose group, protein samples were prepared using six samples, with at least three repetitions.

### 2.7. Data Analysis and Statistics

All data are expressed as means ± SEM unless noted otherwise. After we tested the normality and homogeneity of variance of the data, all body and tissue weight as well as histological data were analyzed via two-way ANOVA (PSNPs dose × sex) followed by Tukey’s post hoc test. The dose differences in the expression of lipogenesis- and lipophagy-related genes were analyzed using Dunnett’s protected least significant difference test. Statistical analyses were conducted using GraphPad Prism (version 8.0.2, GraphPad Software, San Diego, CA, USA). Significance was set at *p* < 0.05.

## 3. Results

### 3.1. Prenatal PSNPs Exposure Led to Low-Birth-Weight Offspring

**Figure 1:**
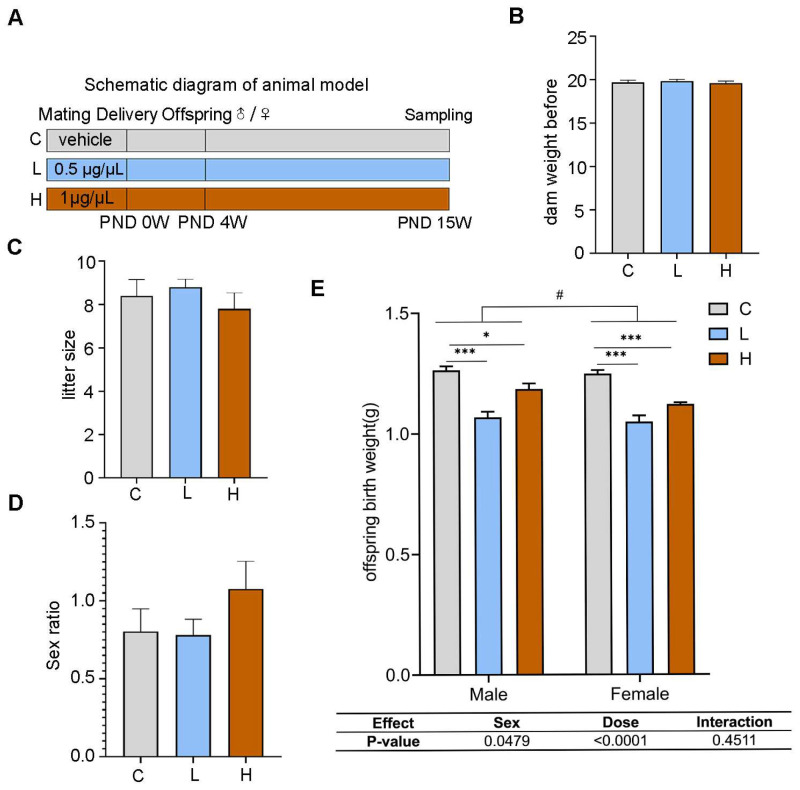
Effect of prenatal PSNPs exposure on outcomes of newborns. (**A**) Animal model; (**B**) body weight before mating; (**C**) number of pups per litter; (**D**) sex ratio of newborn mice per litter; (**E**) birth weight of offspring. *n* = 22–24/group. Compared to the control group, * *p* < 0.05, and *** *p* < 0.001. Data tables show the *p*-values from the two-way ANOVA analyses for the factors ‘Sex’ and ‘Dose’. For comparisons between female and male groups, ^#^ *p* < 0.05.

We first examined the impact of gestational exposure to PSNPs on neonatal progeny. Female C57/BL6J mice were administered a vehicle and differing concentrations of PSNPs (0.5 and 1 μg/μL) throughout pregnancy (Figure 1A), with no variation in dam weight noted before mating (Figure 1B). The results showed that there were no differences in litter size (Figure 1C) or sex ration (Figure 1D) between the groups. Due to the strong correlation between birth weight and long-term metabolic disorders, we assessed the body weights of neonatal offspring mice subjected to prenatal PSNPs exposure. The results showed significantly reduced birth weight for both females and males (Figure 1E; FC: 1.24 ± 0.05 vs. FL: 1.04 ± 0.09 and FH: 1.12 ± 0.03 g; MC: 1.26 ± 0.06 vs. ML: 1.06 ± 0.09 and MH: 1.16 ± 0.06 g) following gestational PSNPs exposure to both low and high doses in comparison to the control groups (*p* < 0.05).

### 3.2. Prenatal PSNPs Exposure Prompted Obesity Development in Adult Offspring

**Figure 2:**
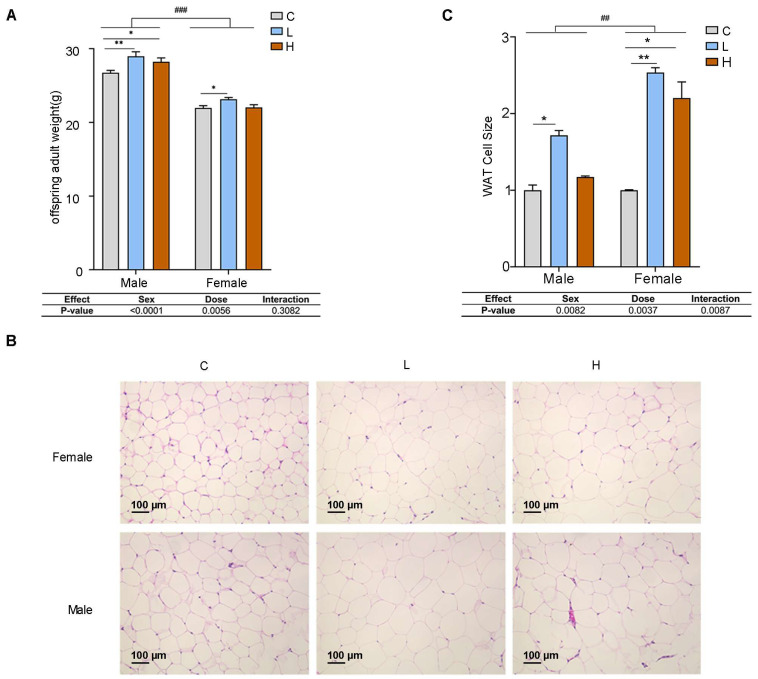
Effect of prenatal PSNPs exposure on the development of obesity in adult offspring. (**A**) Body weight of adult offspring mice (female: *n* = 14–15/group, male: *n* = 11–15/group); (**B**) HE staining of white adipose tissue in adult offspring mice (*n* = 3/group); (**C**) quantification of HE staining of white adipose tissue in adult offspring mice (*n* = 3/group). Compared to the control group, * *p* < 0.05, and ** *p* < 0.01. Data tables show the *p*-values from the two-way ANOVA analyses for the factors ‘Sex’ and ‘Dose’. For comparisons between female and male groups, ^##^ *p* < 0.01, and ^###^ *p* < 0.001.

In order to explore the impact of prenatal PSNPs exposure on the development of obesity in the adult offspring, the offspring mice were fed for up to 15 weeks. The results showed that prenatal PSNPs exposure significantly increased the body weight of both male and female (Figure 2A; MC: 26.81 ± 1.06 vs. ML: 28.94 ± 2.32 and MH: 28.22 ± 1.99 g; FC: 21.99 ± 1.03 vs. FL: 23.21 ± 0.84 and FH: 22.06 ± 1.39 g) adult offspring. White adipose tissue, an essential tissue for energy storage in the body [32], showed enlargement of white adipocytes in the adult offspring mice subject to gestational PSNPs exposure (Figure 2B). Upon quantification, the results also revealed significantly increased cell sizes compared to the control group (Figure 2C; FC: 0.66 ± 0.01 vs. FL: 1.68 ± 0.10 and FH: 1.46 ± 0.34 μm^2^; MC: 0.74 ± 0.12 vs. ML: 1.27 ± 0.11 and MH: 0.87 ± 0.02 μm^2^; *p* < 0.05).

### 3.3. Prenatal PSNPs Exposure Triggered Brown Adipose Tissue Whitening in Adult Offspring Mice

**Figure 3:**
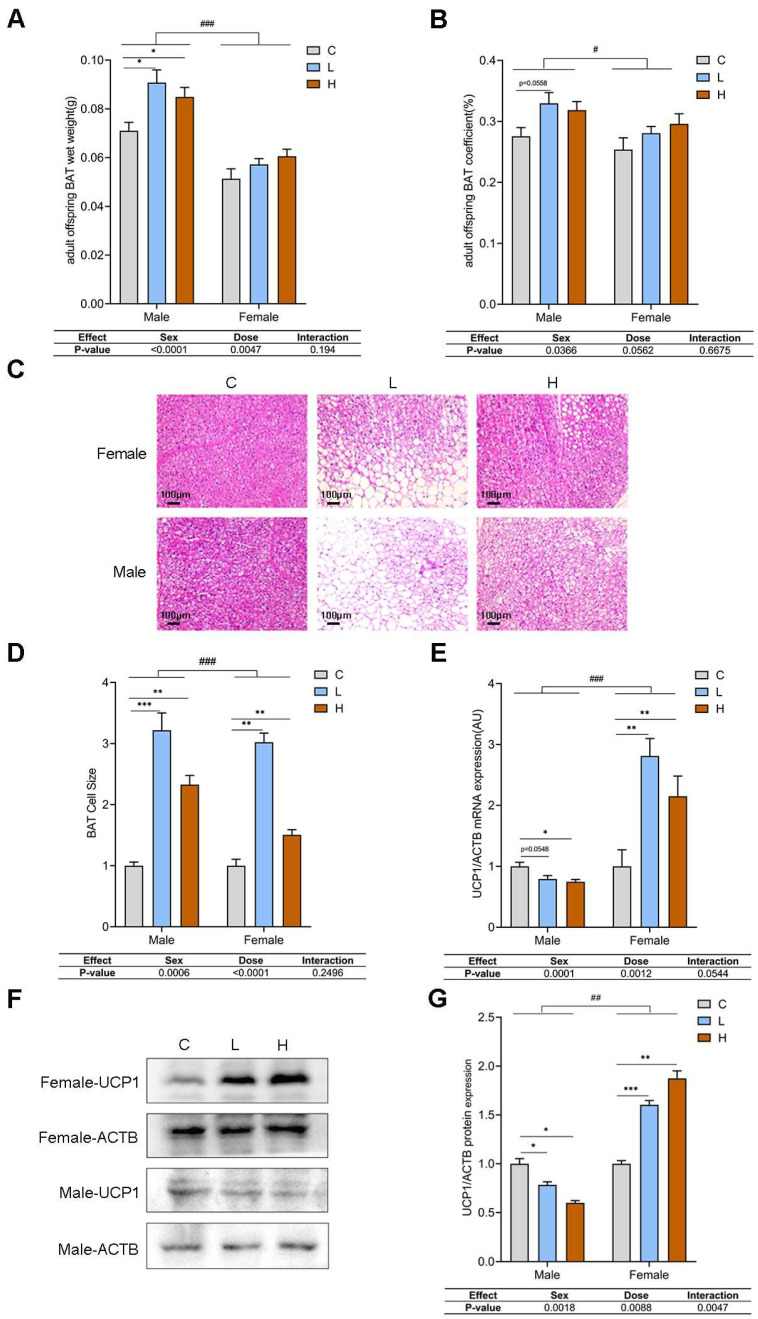
Effect of prenatal PSNPs exposure on BAT whitening and thermogenesis in adult offspring mice. (**A**) Wet weight of BAT in adult offspring mice (female: *n* = 14–15/group, male: *n* = 11–15/group); (**B**) BAT organ coefficient for adult offspring mice (female: *n* = 14–15/group, male: *n* = 11–15/group); (**C**) HE staining of BAT (*n* = 3/group); (**D**) quantification of BAT cell area based on HE staining (*n* = 3/group); (**E**) UCP1 transcription levels in adult offspring mice (*n* = 6/group); (**F**) UCP1 protein expression levels in adult offspring mice (*n* = 6/group); (**G**) quantification of UCP1 protein expression levels in adult offspring mice (*n* = 6/group). Compared to the control group, * *p* < 0.05, ** *p* < 0.01, and *** *p* < 0.001. Data tables show the *p*-values from the two-way ANOVA analyses for the factors ‘Sex’ and ‘Dose’. For comparisons between female and male groups, ^#^ *p* < 0.05, ^##^ *p* < 0.01, and ^###^ *p* < 0.001.

Adipose tissues mainly consist of WAT and BAT, which have distinct characteristics. While WAT serves as a reservoir for energy storage in lipid droplets, BAT metabolizes these lipid droplets to generate heat, with UCP1 regulating its thermogenic energy conversion to heat [33]. The conversion of the lipid reservoir from multilocular to unilocular has been documented in brown adipocytes in the context of obesity; the transition from BAT to WAT is referred to as whitening, which is a significant risk factor for the onset of obesity [34,35,36]. On this basis, we first investigated brown adipose tissue whitening in offspring mice subjected to prenatal PSNPs exposure. The results showed, in adult male offspring mice, significantly increased BAT weight (Figure 3A; MC: 0.071 ± 0.009 vs. ML: 0.091 ± 0.016 and MH: 0.085 ± 0.012 g) (*p* < 0.05) but no significant effect on the organ coefficient (Figure 3B). For adult female offspring mice, prenatal PSNPs exposure had no significant effects on either BAT weight (Figure 3A; FC: 0.051 ± 0.012 vs. FL: 0.057 ± 0.009 and FH: 0.061 ± 0.013 g) or the organ coefficient (Figure 3B). H&E staining (Figure 3C) of the BAT revealed that the brown adipose tissue changed from multilocular small lipid droplets to unilocular large lipid droplets in the offspring of dams exposed to PSNPs compared with the control group. The area of adipocytes in the BAT was also significantly greater than that of the control group (Figure 3D; FC: 0.06 ± 0.01 vs. FL: 0.18 ± 0.02 and FH: 0.09 ± 0.02 μm^2^; MC: 0.07 ± 0.01 vs. ML: 0.22 ± 0.05 and MH: 0.16 ± 0.02 μm^2^). To explore the thermogenic activity of BAT at the molecular level, we analyzed the transcriptional and protein levels of UCP1. The results showed that the mRNA levels (Figure 3E) and protein expression (Figure 3F,G) of UCP1 decreased with a negative dose-dependent relationship in the adult male offspring (*p* < 0.05). However, the mRNA (Figure 3E) and protein (Figure 3F,G) levels of UCP1 were significantly upregulated in the adult female offspring (*p* < 0.05).

### 3.4. Prenatal PSNPs Exposure Enhanced Lipogenesis in Brown Adipose Tissue of Adult Male Offspring Mice

**Figure 4:**
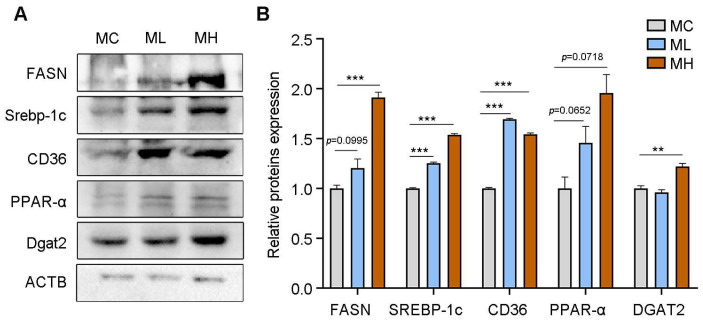
Effect of prenatal PSNPs exposure on lipogenesis: (**A**) protein levels of lipid-synthesis-related genes in brown adipose tissue of adult male offspring mice (*n* = 6/group) and (**B**) quantification of protein levels of lipid-synthesis-related genes in brown adipose tissue of adult male offspring mice (*n* = 6/group). Compared to the control group, ** *p* < 0.01, and *** *p* < 0.001.

For the adult male offspring that showed BAT whitening with impaired thermogenic function, we further investigated the mechanism of BAT whitening from the perspective of lipid metabolism. The results showed that exposure to PSNPs during pregnancy significantly increased the expression of the fatty acid de novo lipogenesis synthesis proteins FASN and SREBP-1c, the fatty acid transport protein CD36, and the triglyceride synthesis enzyme DGAT2 in adult male offspring mice (Figure 4A,B; *p* < 0.05). Notably, the expression level of the fatty-acid-degrading protein PPAR-α showed a downward trend, but this trend was not statistically significant (Figure 4A,B; *p* < 0.05).

### 3.5. Prenatal PSNPs Exposure Inhibited Lipophagy in Brown Adipose Tissue of Adult Male Offspring Mice

**Figure 5:**
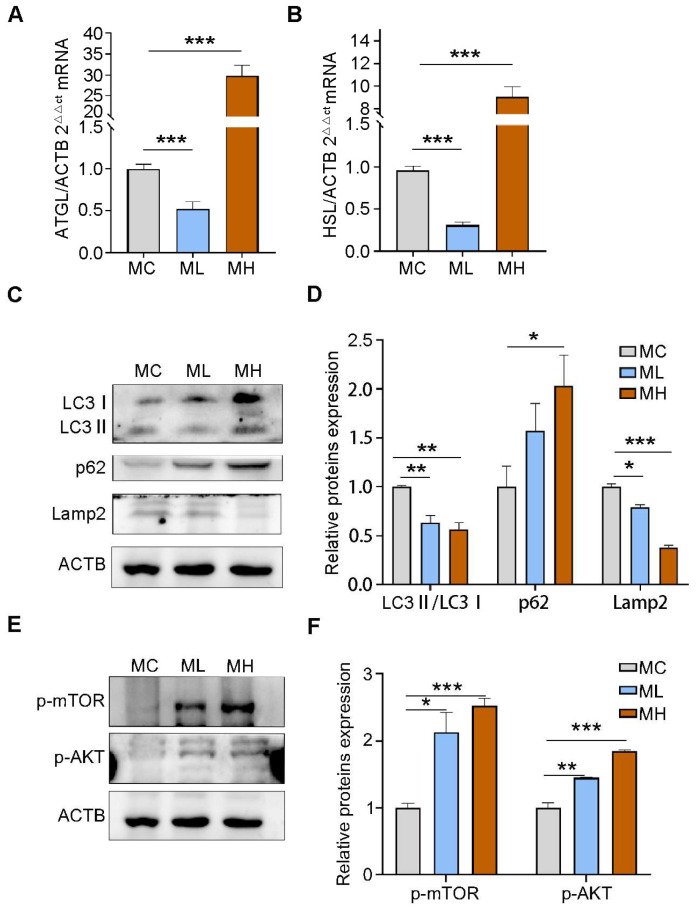
Effect of prenatal PSNPs exposure on lipophagy in BAT of adult male offspring mice. (**A**) ATGL mRNA levels in the brown adipose tissue of adult male offspring mice; (**B**) HSL mRNA levels in the brown adipose tissue of adult male offspring mice; (**C**) protein expression levels of lipophagy-related genes; (**D**) quantification of protein expression levels of lipophagy-related genes; (**E**) protein levels of mTOR and AKT in brown adipose tissue of adult male offspring mice; (**F**) quantification of mTOR and AKT protein levels in brown adipose tissue of adult male offspring mice. *n* = 6/group. Compared to the control group, * *p* < 0.05, ** *p* < 0.01, and *** *p* < 0.001.

Lipophagy refers to the specific degradation of lipid droplets (LDs) via the autophagic pathway, which is critical in regulating intracellular lipid metabolism [37]. In the BAT of offspring male mice, we initially assessed the transcription levels of ATGL and HSL, which are the essential lipolytic genes. The results showed that prenatal exposure to high doses of PSNPs significantly upregulated the transcription levels of ATGL and HSL, whereas prenatal exposure to low doses of PSNPs markedly decreased their expression compared with the control group (Figure 5A,B), indicating the deregulated breakdown of lipid droplets. We further analyzed the protein expression of lipophagy-related genes in these mice. The results showed that the protein levels of LC3II/LC3I and Lamp2 were significantly downregulated, and the protein levels of P62 were significantly upregulated (Figure 5C,D; *p* < 0.05), in the offspring of dams exposed to PSNPs, indicating the inhibition of lipophagy. The AKT/mTOR pathway is pivotal in regulating lipophagy [38]. Activation of this pathway leads to increased AKT and mTOR phosphorylation levels, inhibiting the lipophagic process. We assessed the protein levels of AKT and mTOR to investigate whether the molecular mechanism of prenatal-PSNP-exposure-inhibited lipophagy is associated with the AKT/mTOR pathway. As expected, following prenatal PSNP exposure, the protein expression levels of p-mTOR and p-AKT were markedly increased (Figure 5E,F; *p* < 0.05). The results noted above indicate that lipophagic inhibition mediates BAT whiteninginduced by prenatal PSNPs exposure via the AKT/mTOR pathway.

## 4. Discussion

With plastic pollution aggravating over time, the latent health hazards arising from micro- and nanoplasticshave gained increasing attention. Simultaneously, obesity has become a common concern, and BAT whitening is closely related to the development of obesity. Thus, in the current investigation, we investigated the impact of gestational exposure to airborne PSNPs on the whitening of BAT in offspring and further assessed the underlying mechanisms. This study confirmed that gestational exposure to PSNPs led to obesity in adult offspring. More importantly, we discovered that maternal exposure to PSNPs during pregnancy initiated BAT whitening in both female and male offspring, with only male progeny exhibiting reduced thermogenic activity. Furthermore, our findings revealed that maternal exposure to PSNPs significantly upregulated genes associated with lipogenesis and the inhibition of lipophagy via AKT/mTOR pathway activation, contributing to the whitening of BAT.

Obesity is presently one of the most significant global health issues, elevating the risk of diabetes, hypertension, fatty liver disease, cardiovascular ailments, and cancer [39,40,41]. Moreover, the population of overweight and obese individuals continues to surge swiftly across the globe [42], which intensifies the necessity of identifying the risk factors associated with obesity. Recently, alongside evident dietary habits and physical activity, an increasing number of reports have revealed that early-life exposure to environmental pollutants contributes to the development of obesity [43,44,45,46]. In this study, we found that gestational exposure to airborne PSNPs induced the development of obesity in adult offspring, a result consistent with the results of previous studies [20,47] indicating that early-life exposure to nanoplastics hastens the emergence of obesity. What is more, Jeong et al. [20] reported that maternal exposure to a dose of 10 μg/day during gestation and lactation through the digestive route has no effect on body weight gain among mouse offspring. In our study, we found that gestational exposure to PSNPs at a dose under 1 μg/μL (about 10.70 μg/day) via inhalation markedly increased the body weight of and fat in adult offspring. Combined with the daily microplastic exposure amounting to approximately 0.012 g/day for humans [48], these research findings call for increased attention to the potential impacts of microplastics and nanoplastics on human health, especially via the respiratory exposure route.

The physicochemical properties of nanoplastics, especially particle size, play a vital role in evaluating nanoplastics’ potential toxic effects on offspring during gestational exposure. In this study, 80 nm particles were employed to evaluate transgenerational toxicity. A previous study [21] demonstrated that 80 nm PSNPs predominantly concentrated in the lungs of dams, and no notable signal was detected in the placenta or fetus. And Tian et al. [49], after using 3, 13, and 32 nm gold nanoparticles for tail vein injection in murine pregnancy, reported that the concentration of 3 nm gold nanoparticles was much higher than that of 13 nm gold nanoparticles in the fetal tissue of intrauterine inflammatory mice, while 32 nm gold nanoparticles could not cross the placental barrier into the offspring. The difference in the results is probably due to the fact that we used larger particles that are less efficient in crossing the blood–air barrier as well as because, in this study, the entire gestational period was 21 days, which is not enough time for significant extrapulmonary transfer to occur. Therefore, in this study, the toxic effects on the offspring may have been caused by damage in the parental generation, rather than direct interaction with adipose tissue of the offspring causing obesity, a factor that needs to be investigated in further studies.

To further investigate the underlying mechanism through which prenatal PSNPs exposure induces obesity, considering that BAT whitening prompts abnormal thermogenesis, increasing the probability of obesity [50], we initially documented the phenomenon of BAT whitening in male offspring subjected to prenatal PSNPs exposure. Nicotine, during pregnancy and lactation, reduces BAT vascularization during early development and BAT phenotype characteristics in adulthood by downregulating the activity of the PGC-1α-UCP1 signaling pathway, which may be a reason for nicotine-induced obesity in female offspring, which is consistent with the results of our study [26]. Together with our study, evidence indicates that BAT whitening may mediate obesity induced by prenatal exposure to environmental toxins and provide a novel target for addressing obesity. Although both female and male offspring exhibited browning of white adipose tissue, we observed that only the male offspring showed a significant decrease in UCP1 expression—a key marker of thermogenic capacity. This differential response was a critical factor in our decision to focus our subsequent mechanistic analyses on male tissues. It is well established that estrogen plays a protective role in regulating adipose tissue distribution and metabolism [51]. We hypothesize that in female offspring, estrogen may exert a compensatory effect, maintaining thermogenic function and thereby mitigating the reduction in UCP1 levels observed in males. Additionally, the compensatory mechanism likely obscures the direct effects of PSNPs exposure on thermogenesis in females, a facet worthy of further exploration in future studies. Thus, male offspring were selected in order to clearly delineate the underlying mechanisms associated with impaired thermogenic function.

Many studies have suggested there is an intimate linkage between lipogenesis and BAT whitening. Lipogenesis occurs due to increased de novo lipogenesis (DNL) [52] and enhanced triglyceride (TG) synthesis [53]. Our research demonstrates that prenatal exposure to PSNPs promotes lipid accumulation in the brown adipose tissue of adult male offspring by upregulating the protein expression involved in the de novo fatty acid synthesis pathway, including FASN and SREBP-1c, the fatty acid transporter CD36, and the triglyceride synthesis enzyme DGAT2. A recent study reported that DNL in BAT determines BAT whitening in response to thermoneutral housing [54]. Furthermore, due to the lack of DGAT2, triacylglycerol stores and lipid droplets are absent in brown adipocytes in mice [55]. Moreover, CD36 promotes glucose transport and lipogenesis by increasing the quantity of lipid droplets [56]. In accordance with our study, these results demonstrate the important role of lipogenesis in BAT whitening

In lipid metabolism, autophagy has been found to regulate both the biosynthesis and degradation of LDs, and this process is referred to as lipophagy [57]. Lipolysis, a conventional lipase-driven process regulating LD turnover, targets larger-sized LDs to generate smaller-sized LDs [58]. The cytosolic lipases ATGL and HSL contribute to LDs’ mobilization [59], which can then be catabolized by lipophagy. This mechanism is crucial in regulating the biology of brown adipose tissue [60]. In our study, we found that prenatal PSNPs exposure significantly upregulated the expression of ATGL and HSL, suggesting that larger LDs are broken down into smaller ones. The results also showed increased LC3 and p62 and decreased LAMP2, indicating that the activation of autophagy was inhibited. Wang et al. found that Bisphenol F could induce hepatic lipid accumulation by inhibiting lipopagy, a result that is consistent with our study [61], while activating lipophagy could restore the degradation of lipid droplets and inhibit fat accumulation [62,63]. We also found that upregulations in the lipophagic signaling pathways of mTOR and AKT, results that are consistent with those of a previous study [64]. Consequently, the results of our study indicate that regulating lipophagy via suitable targets to counteract the whitening of brown adipose tissue may represent a promising therapeutic target for obesity.

## 5. Limitations of This Study

In the real-world environment, nanoplastic particles exhibit an extensive array of diverse properties. Nevertheless, the nanoplastics employed in this study were predominantly spherical in form. This characteristic may not accurately mirror the complex and transformed nanoplastics that pregnant individuals are exposed to in actual scenarios, thus limiting the ability to directly extrapolate our findings to actual environmental health risks. Additionally, the exposure frequency in this study was set at three times a week. In contrast, human exposure to nanoplastics in everyday life is likely to be more variable and continuous. The fixed exposure frequency in our study cannot account for the dynamic nature of human exposure, which may have led to an over- or under-estimation of the actual impact of gestational nanoplastic exposure on offspring obesity. As a consequence, the ability to translate our experimental results into real-world human health implications is somewhat restricted.

## 6. Conclusions

The findings in the present study suggest that exposure to PSNPs during pregnancy contributes to the development of obesity in offspring by inducing the whitening of BAT. Further study revealed that the process of the whitening of BAT induced by prenatal PSNPs exposure was triggered by enhanced lipogenesis and the inhibition of lipophagy, which disrupts normal lipid metabolism and the thermogenic function of BAT. Our study provides a novel insight into the metabolic risks associated with gestational PSNPs exposure, highlighting the potential long-term consequences of environmental nanoparticle exposure during critical periods of development. And the genes and pathways linked to BAT whitening uncovered here may provide potentially novel targets for the prevention and management of obesity by counteracting the whitening of brown adipose tissue. Modulating autophagy via using suitable targets to counteract the whitening of brown adipose tissue may represent a promising therapeutic target for obesity and related metabolic disorders.

## Figures and Tables

**Table 1:** Primer sequence table.

Gene	Forward Primer (5–3′)	Reverse Primer (5–3′)
β-actin	TGAACGGGAAGCTCACTGG	TCCACCACCCTGTTGCTGTA
ATGL	CAGAGATGGACTTCGATTCCTT	CAGGTGCTCTAGAATTCGATCT
HSL	CTCACAGTTACCATCTCACCTC	GATTTTGCCAGGCTGTTGAGTA
UCP-1	CACGGGGACCTACAATGCTT	CAGGAGTGTGGTGCAAAACC

**Table 2:** Antibody dilution rate.

Protein	Source	Dilution Rate	Brand	Art. No.
LC3	Rabbit	1:5000	Proteintech	14600
P62	Rabbit	1:1000	Proteintech	18420
p-mTOR	Rabbit	1:2000	Abways	CY6571
p-AKT	Rabbit	1:2000	Abways	CY6569
Lamp2	Rabbit	1:2000	Abways	CY5518
PPAR-α	Rabbit	1:1000	Proteintech	15540
SREBP1	Rabbit	1:5000	Abcam	ab28481
FASN	Rabbit	1:1000	Cell Signaling	3180S
CD36	Rabbit	1:2000	proteintech	18836
DGAT2	Rabbit	1:2000	Immunoway	YN0642
UCP-1	Rabbit	1:1000	Cell Signaling	14670S
β-actin	Mouse	1:20,000	Immunoway	YM3028

## Data Availability

The datasets supporting the conclusions of this article are included within the article and can be retrieved from the corresponding author upon reasonable request.
